# New﻿ extreme morphologies as exemplified by 100 million-year-old lacewing larvae

**DOI:** 10.1038/s41598-021-99480-w

**Published:** 2021-10-14

**Authors:** Joachim T. Haug, Viktor Baranov, Patrick Müller, Carolin Haug

**Affiliations:** 1 Ludwig-Maximilians-Universität München (LMU Munich), Großhaderner Str. 2, 82152 Planegg-Martinsried, Germany; 2 GeoBio-Center at LMU, Richard-Wagner-Str. 10, 80333 München, Germany; 3 Zweibrücken, Germany

**Keywords:** Entomology, Palaeontology

## Abstract

Larvae of the group Holometabola (beetles, wasps, flies, moths and others) differ significantly in their morphology from their corresponding adults. In most larvae, appendages and other structures protruding from the body (antennae, palps, legs, trunk processes) appear less elongate than in their corresponding adults, providing the impression that these larvae are restricted to a certain degree in developing more elongate structures. We provide here numerous counterexamples of larvae of lacewings (Neuroptera). These include different forms of elongated antennae, mandibles, maxillae, labial palps, legs, trunk processes and neck regions. Most of these examples are larvae preserved in different types of 100 million-year-old amber. The longest neck region was found in an extant specimen. All these examples demonstrate that certain branches of Neuroptera indeed had larval forms that possessed strongly elongated structures. Hence there is no principal constraint that hinders holometabolan larvae to develop such structures.

## Introduction

The dominating part of the modern day terrestrial fauna is represented by the group Holometabola with its four major hyper-diverse lineages, each with far more than 100,000 formally described species^1^: Hymenoptera (wasps, bees, ants), Coleoptera (beetles, weevils), Lepidoptera (moths, butterflies) and Diptera (flies, mosquitoes, midges, gnats). Besides these, there are additional holometabolan lineages that have “only” some thousands of species such as Trichoptera (caddisflies), Neuroptera (lacewings and relatives), or Siphonaptera (fleas)^1,2^. The enormous evolutionary and ecological success of Holometabola has been attributed to the niche differentiation between the adult and the often highly aberrant appearing larva^3^.

Despite the larvae playing a major role for the diversification of holometabolans, it is often not easy to identify a larva to a species. This seems to have somehow led to the impression that adults are more diverse concerning their morphological variation than larvae. This idea may have additionally been reinforced by the work of Ernst H.P.A. Haeckel (see discussion by de Beer^4^, who criticizes Haeckel by showing examples of holometabolan insects in which the adults are not easily distinguishable while the larvae clearly are). When looking outside Insecta into some of the ingroups of Euarthropoda, such as Cirripedia (barnacles and their relatives), we indeed find good examples for highly morphologically derived adults, but strongly conserved or stereotypic larval morphologies^5^. It also has been suggested that holometabolan larvae are something like early hatched embryos, hence possessing a type of “not-yet-ready” morphology^6^. Indeed quite recently, the ancestral type of larva in Holometabola has been reconstructed as a rather simple-organised one; many structures such as eyes, antennae, mouthparts, and locomotory legs are usually considered to be reduced or simplified^7,8^. All these notions indirectly imply that holometabolan larvae are to a certain degree constrained in their possibilities to evolve morphologically diverse forms. Indeed, numerous larvae of holometabolans have a rather simple, almost worm-like body, as for example in caterpillars, grubs, or maggots^1^.

Here we demonstrate that many larvae of lacewings deviate significantly from this seemingly general pattern. Especially fossil resins (= amber) known from the Cretaceous (between 130–100 million years old) have provided larvae with different structures of hypertrophied size. This includes larvae with super-sized mouthparts, unusual armament on the trunk, and long “necks” i.e. the region directly posterior to the head capsule. These unusual forms illustrate the large diversity of shapes existing in larvae of the group Holometabola. With this, they extend our understanding of the diversification of Neuroptera in the Mesozoic. Herein, we review known forms, demonstrating their morphological exceptionality, and report new, highly unusual lacewing larvae with hypertrophied mouthparts preserved in 100 million-year-old Cretaceous amber from Myanmar.

## Results and discussion

### General comment on morphology of lacewing larvae

All lacewing larvae have highly derived venom-injecting, piercing-sucking mouthparts. Each upper jaw (mandible) is conjoined with its corresponding lower jaw (maxilla) to form a so-called “stylet”^9,10^. These can be formed in various ways, in some of the cases presented here in a rather unusual manner.

### New larvae with prominent straight stylets

**Figure 1:**
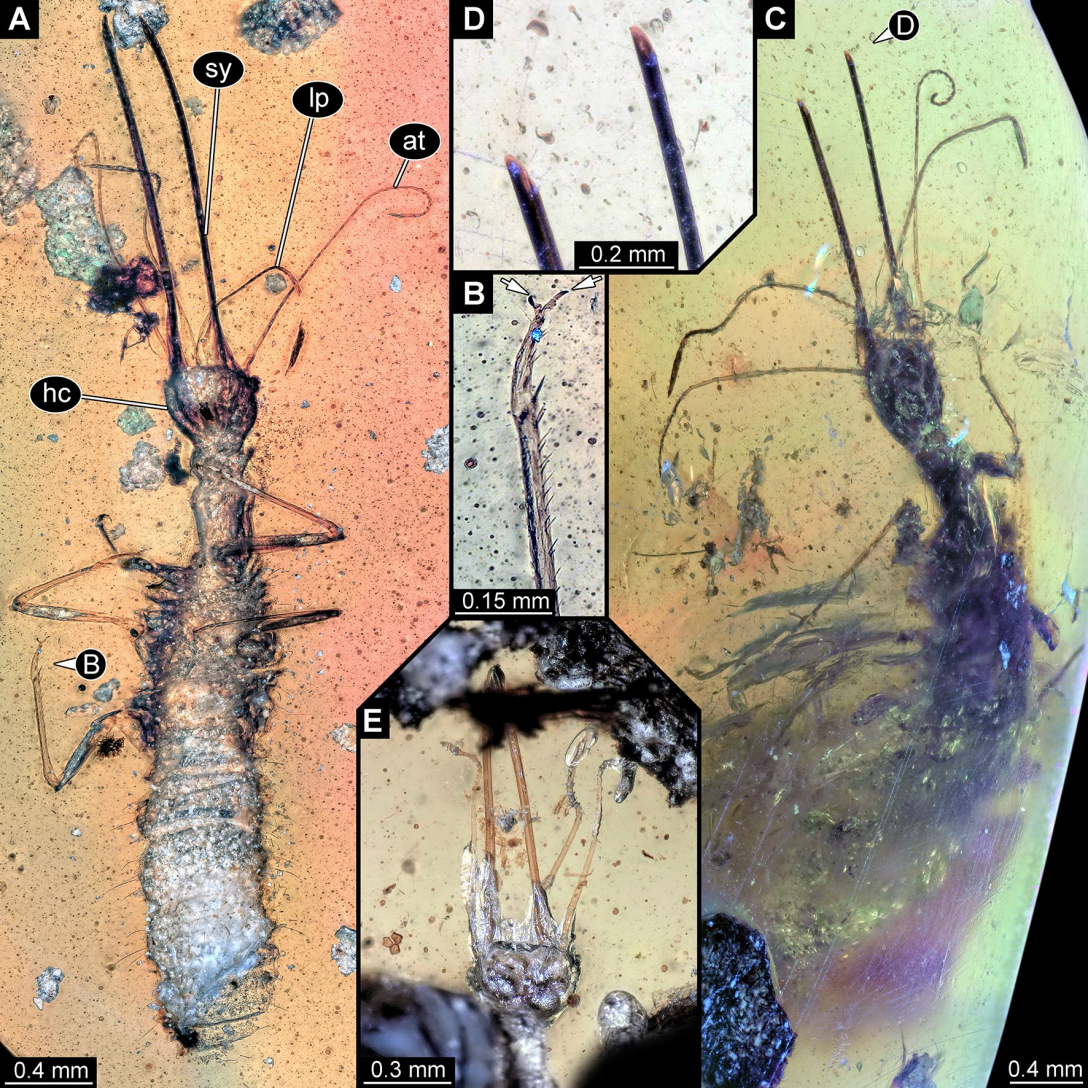
Lacewing larvae of the “Supersting”-type in amber from Myanmar; all with elongate appendages. (**A**, **B**) Specimen BUB 3133, (**A**) Overview in ventral view. (**B**) Close-up of tip of hind leg; arrows mark tarsal claws. (**C**, **D**) Specimen PED 0316. (**C**) Overview in dorsal view. (**D**) Close-up of broke-off tips of preserved stylets. (**E**) Specimen BUB 3348a. *at* antennule, *hc* head capsule, *lp* labial palp, *sy* stylet.

Three new specimens appear very similar, but incomplete in different aspects. One specimen lacks the trunk end (Fig. 1A,B), one has large parts of the trunk concealed by irregularities of the amber (Fig. 1C) and lacks the distal part of the stylets (Fig. 1D), and one is largely obscured by dirt particles (Fig. 1E), providing mostly access to the head.

All three have very prominent mouthparts forming functional sucking stylets. Together with a sclerotised neck region, this immediately identifies them as a larvae of lacewings, i.e., a representative of the group Neuroptera. Most lacewing larvae are characterised by curved, counteracting stylets (see discussion in Haug et al.^11,12^ about advantages of this morphology). More or less straight stylets with forward facing tips occur only in larvae of few lineages^11,12^.

A fossil lacewing larva closely resembling new fossils was recently reported^11^ and given the nickname “Supersting”, referring to its extremely long stylets. All the new specimens have very elongated stylets, mostly straight, but forming a slight S-shape. They also have very long antennae with numerous elements (antennomeres), as well as very long labial palps, strongly resembling the antennae, and reaching about 90% of the antenna length. The new larvae might either be closely related to the known “Supersting” larva or even be conspecific with it, potentially representing later instars of this species. This interpretation would be compatible with the larger size, as well as the even more elongate mouthparts of the new specimens, as compared to the original “Supersting” specimen^11^. It was so far not possible to clearly identify a closer relationship of the original “Supersting” larva within Neuroptera. Hence the interpretation of the new larvae as representatives of Neuroptera is beyond doubt, yet a closer relationship to any modern group cannot be easily inferred.

### New larva with prominent, simple curved stylets

**Figure 2:**
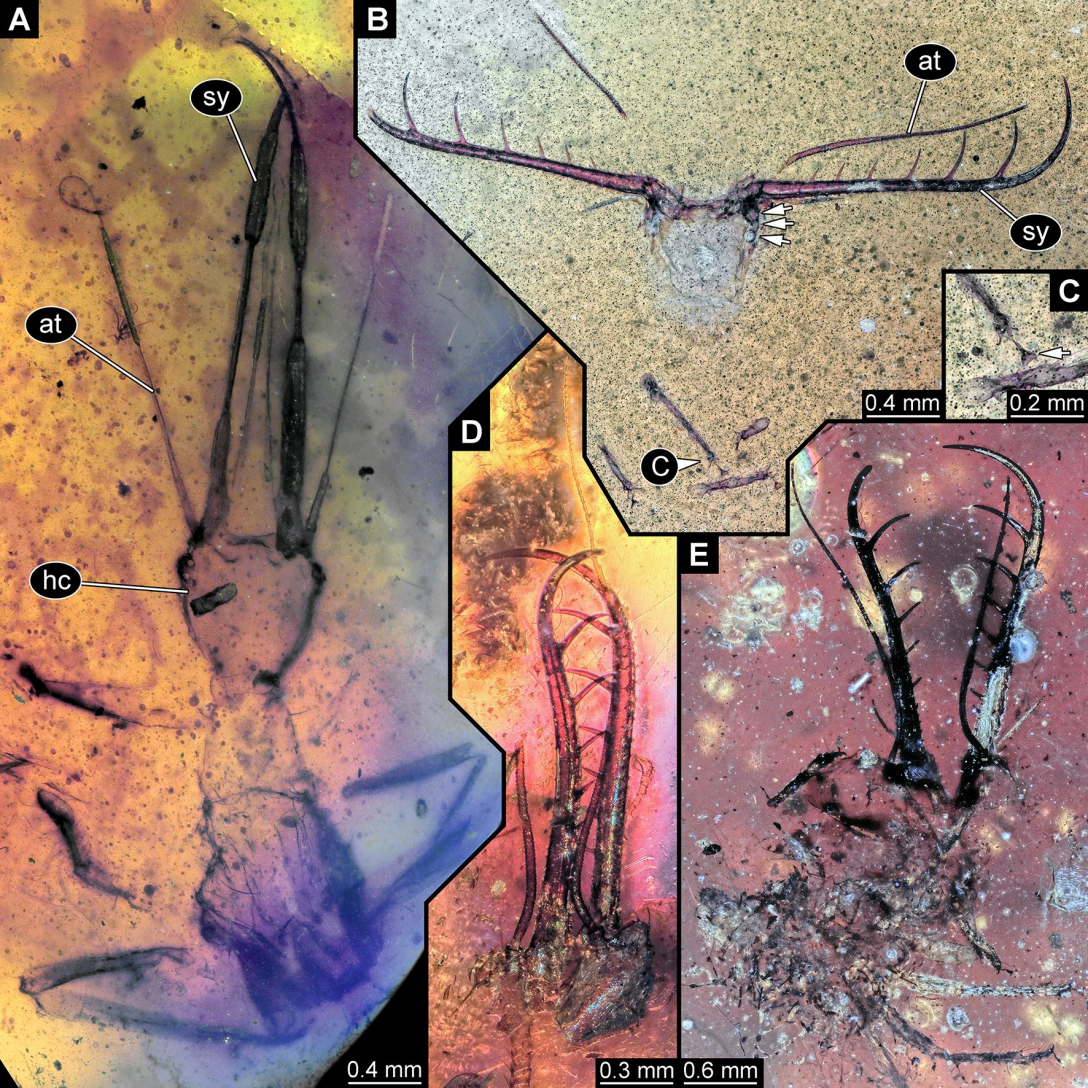
Lacewing larvae in amber from Myanmar; all with elongate, curved stylets. (**A**) PED 0188, larva possibly closely related to Chrysopidae. (**B**–**E**) Larvae with stylets bearing six prominent teeth. (**B**, **C**) PED 0361. (**B**) Overview; arrows mark stemmata. (**C**) Close-up of walking legs; arrow marks empodium. (**D**) PED 0088. (**E**) PED 0112. *at* antennule, *hc* head capsule, *sy* stylet.

One new specimen (Fig. 2A) has very long elongate stylets, which unlike in the previous specimens are gently curved towards the tip. In some other aspects, it also resembles the “Supersting”-type larvae: antennae and palps are rather long, although they appear slightly shorter. Also the neck region is prominent. Most of the trunk is not preserved. Overall, the specimen resembles specimens that have been interpreted as larvae of species related to modern green lacewings (Chrysopidae;^13^, their upper fig. on p. 95;^14^, his upper fig. on p. 384). The overall morphology is also compatible with an interpretation of Neuroptera, and a closer relationship to Chrysopidae seems at least possible. Yet, many details that could further corroborate such an interpretation are not accessible in the new specimen and also difficult to discern in the already known specimens.

### New larvae with prominent toothed curved stylets

Three new specimens have extremely prominent stylets with numerous, also prominent teeth (Fig. 2B–E). Only the stylets are well preserved, all the remaining parts appear crumpled and almost translucent. Most likely, these specimens are exuviae explaining this condition. Based on the mandibles, these fossils are distantly similar, or at least comparable to the larva with the nickname “Superfang” described by Haug et al.^12^ and also to a larva formally described as *Cladofer huangi*^15^. The head is quite narrow for the long stylets, narrower than that of *C. huangi*^15^, quite similar to that of the original “Superfang” larva^12^.

The new larvae are different to the two other larvae in the exact arrangement of the teeth. The teeth are longer than in the original “Superfang” larva, which has eleven, but smaller teeth^12^. The new larvae have only six teeth, in this aspect they are more comparable to the single specimen of *C. huangi*^15^, although the latter has even one tooth more.

The new specimens also provide some details not accessible (and hence not comparable) in the original “Superfang” larva^12^ and *C. huangi*^15^, as e.g. the tips of the trunk appendages (“legs”), which bear prominent attachment structures (empodia; Fig. 2C). Among modern representatives of Myrmeleontiformia (antlion-like lacewings), larvae either bear teeth in the mandibles (as larvae of Nymphidae, Ascalaphidae, Myrmeleontidae, to a certain degree also Nemopteridae; see discussion in^16^ about ontogenetic reduction of such teeth), or empodia (Psychopsidae), or neither nor (certain larvae of Nemopteridae). Few fossils from the Cretaceous are known to have both^15,17^. These and *C. huangi*^15^ have been interpreted as very early derivatives of the group Myrmeleontiformia^15,18^. The original “Superfang” larva^12^ and the new larvae may therefore be also a part of this very early radiation of Myrmeleontiformia.

### The morphology of the ancestral holometabolan larva

The ground pattern of the ancestral holometabolan larva has been heavily debated^19,20^. Labandeira^21^ suggested that it was eruciform, hence strongly resembling a caterpillar in many aspects, as in larvae of Lepidoptera, non-parasitoid larvae of Hymenoptera, and non-aquatic larvae of Mecoptera^22^. The oldest fossils of holometabolan larvae indicate that the case is less simple as these combine certain aspects of the eruciform morphology with characters of the campodeiform larval type, i.e., highly mobile larvae of many different groups, including for example predatory beetle larvae. In particular, these traits include longer legs and a slightly dorso-ventrally flattened trunk^23^.

A recent reconstruction of the ancestral holometabolan larva based on a phylogenetic framework again suggested a rather eruciform morphology, yet without leglets or other appendage-like structures on the abdomen segments (^8^, their Fig. 2). Many of the current reconstructions^8,24^ do agree on the following regarding the ground pattern of the holometabolan larvae: (1) the ancestral holometabolan larva was an elongate organism; (2) its head bore no compound eyes, but stemmata (but see^8^, their Fig. 2); (3) the antennae were rather short, consisting of only few elements (antennomeres); (4) the mouthparts are of a cutting-grinding type; (5) palps of mouthparts are rather short, consisting of only few elements (possibly 3–5); (6) the locomotory appendages, i.e. the legs, are relatively short in comparison to the body length; (7) in case that appendages on the abdomen were present, these were shorter than the locomotory appendages; (8) no dorsal processes were present. These assumptions are backed up by both phylogenetic reconstructions^8,24^ and available fossils^23,25^.

### Deviations from the ancestral state

Evolutionary processes lead to quite a diversity of derived morphologies of holometabolan larvae in the various lineages. Yet, it appears that especially larvae of lacewings have heavily deviated from the ancestral larval morphology, resulting in extreme superlatives^11,12,15,26,27^. Starting with the functional aspects, all lacewing larvae have highly derived venom-injecting, piercing-sucking mouthparts (“stylets”). This is already unusual, as in many other lineages larvae retain rather simple, cutting-grinding type of mouthparts^24^.

Few other examples of derived mouthparts in holometabolan larvae are known. Some highly derived beetle lineages appear to have likewise derived mouthparts already in the larval stage (see^28^ and references therein). Additionally, stylet-like structures do occur in the form of the vertically-articulated mouth hooks in the early branches of the group Brachycera, namely within Orthorrhapha^29^.

Other aspects are more of a quantitative nature, i.e. length of certain structures or number of elements of structures of some lacewing larvae are, mostly, unparalleled among other holometabolan larvae. This includes the following examples:

1. Antennae
  The first appendage (appendage of the first post-ocular segment, antennula in other groups) is usually very short in holometabolan larvae and consists of only very few elements (antennomeres; see above). The antenna of the new “Supersting-type” larvae is very elongate (Fig. 3A), about 30% of the main body length and subdivided into about 30 elements. This exceeds corresponding values in most other known larvae of Insecta. Some fossil larvae, generally interpreted as green lacewings (Chrysopidae), have similarly long antennae, but prove difficult to measure concerning body length (^13^, their upper fig. on p. 95;^14^, his upper fig. on p. 384). In this aspect, lacewing larvae are in fact less extreme than some beetle larvae. Some larval stages of the beetle ingroup Scirtidae possess antennae as long as the main body (^30^, their Fig. 16;^31^, his Fig. 29).

**Figure 3:**
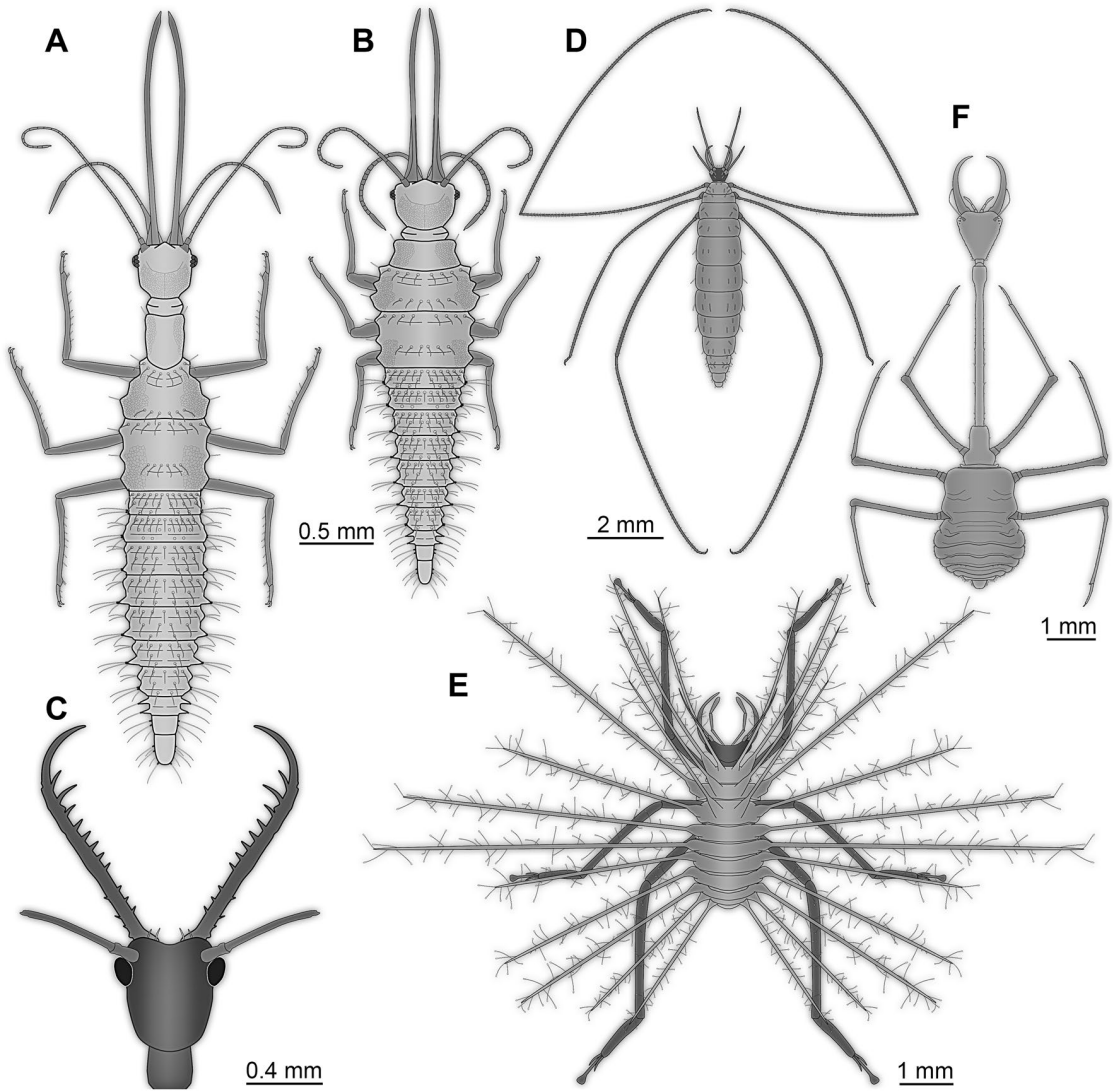
Further lacewing larvae with extreme morphologies. (**A**–**E**) Larvae from Myanmar amber. (**A**, **B**) “Supersting”-type. (**A**) Based on specimen BUB 3133 (see Fig. 1A). (**B**) Original “Supersting” larva^11^. (**C**) Head of “Superfang” larva^12^. (**D**) Larva of *Pedanoptera arachnophila* (simplified from^26^). (**E**) Larva of *Hallucinochrysa diogenesi* (simplified from^36,37^). (**F**) Extant larva of *Necrophylus*^27^.
2. Mandibles
  As all representatives of Insecta lack a well developed appendage on post-ocular segment 2 (intercalary segment;^22,32^), the next to be discussed structure is the mandible, the appendage of post-ocular segment 3. It is often not easily recognised in larvae as it is covered by other structures. Yet, in quite some larvae the mandibles are forward-projecting and rather large as in many representatives of beetles (Coleoptera) and snake flies (Raphidioptera). Lacewing larvae are clearly on the extreme end of the spectrum when it comes to the relative length of the mandibles in larvae of Insecta; they are only comparable in this aspect to some adult forms with likewise extreme morphologies^33^.
  Again one of the new “Supersting”-type larvae sets the scale here. The mandible is even longer than the antenna (Fig. 3A), almost 50% of the (estimated) main body length, hence about one third of the total length. Already the original “Supersting” larva was extreme (Fig. 3B), only topped by few stage 1 lacewing larvae where the trunk has not yet fully expanded^11^, but in one of the new larvae the mandible is even more elongated.
  There are additional extreme cases of extremely long mandibles, though not as long as in “Supersting”, but with different morphology. The mandibles of the “Supersting” larvae are of the “straight” type, piercing stylets directed forward. Among curved mandibles, very elongate forms are known. Larvae generally interpreted as representatives of green lacewings (Chrysopidae) are roughly reaching the relative length of the mandibles of the “Supersting” (Fig. 2A;^13^, their upper fig. on p. 95;^14^, his upper fig. on p. 384).
  Another extreme type of neuropteran larvae with long, curved mandibles differs from the others by possessing numerous prominent teeth, the mandibles of the “Superfang”-type larvae (Fig. 3C;^12^). These mandibles in principle resemble those of larvae of antlions and owlflies, yet, the head and body of these fossil larvae is much more slender than those of the latter. Indeed, many of these fossils appear even more extreme concerning relative mandible length as the trunks appear rather short. Most likely these are exuviae (see above), hence the true ratio is not as extreme, yet in modern-day lacewing larvae similar mandibles appear to have a functional coupling to a relatively broader head^12,34^; hence the “Superfang”-type larvae are superlative concerning their ratio of mandible length to maximum width with toothed mandibles. Also some other lacewing larvae from the Cretaceous reach almost such extreme ratios. All these rather slender larvae seem to represent offshoots of the early lineage of the antlion-like lacewings^12,15^.
3. Maxilla
  As the maxilla (appendage of post-ocular segment 4) in lacewing larvae is functionally tightly coupled to the mandible, all examples discussed above with very long mandibles will also have similarly long maxillae.
4. Labium
  The labium (medially conjoined appendages of post-ocular segment 5) is in many representatives of Insecta rather indistinct^22^. The distal parts (palps) are usually short and consist of only three elements. Also in this case, the new “Supersting”-type larvae are exceptional. The labial palps are as long as the very long antennae (see above) and subdivided into an usually high number of elements. Also here the possible larvae of green lacewings reach comparable lengths (Fig. 2A;^13^, their upper fig. on p. 95;^14^, his upper fig. on p. 384). The exact number of elements is difficult to discern in all known specimens.
5. Thorax appendages (“legs”)
  While many of the above mentioned larvae have relatively long legs (appendages of post-ocular segments 6–8) compared to many other holometabolan larvae and only similar to certain adults (e.g., of crane flies;^35^), all larvae are topped by the quite weird-appearing larva of *Pedanoptera arachnophila* Liu, Zhang, Winterton, Breitkreuz and Engel, 2016 (Fig. 3D;^26^). Thorax appendages 1 and 3 (foreleg and hindleg) of this larva are more than twice as long as the main body (^26^; ^14^, his upper fig on p. 386), very slender and thin. The length to width ratios of the individual leg elements are unparalleled. Though other, supposedly closer related, forms reach similar ratios of leg to body length^36,37^, the legs still appear less extremely elongated, as the length-to-width ratios of the individual leg elements are less extreme.
  Larvae of thread-winged lacewings (Crocinae) also have quite elongate legs (recent overviews in^27,38^). However, the leg-to-body-length ratio is not as extreme in these, as these larvae have a strongly elongated neck region, leading to a much longer body. This shows a certain limitation of comparing relative lengths to reveal extreme forms, as in this case both dimensions forming the ratio become more extreme.
6. Abdomen processes
  Many holometabolan larvae possess dorsal processes on the abdomen segments. This includes various caterpillars of butterflies (Lepidoptera), of scorpion flies (Mecoptera), different larvae of beetles (Coleoptera), but also many lacewing larvae. Many of these processes in lacewing larvae seem to be involved in forming a camouflaging cloak^39,40^. In extreme cases, these processes can clearly exceed the main body length. Comparable lengths seem not to be reached by other holometabolan larvae (Fig. 3E;^36,37^).
7. Elongated neck region
  Holometabolan larvae often possess very evenly-sized segments (at least in the trunk region). Many lacewing larvae possess a distinct sclerite in the neck region between the head and the pronotum. In some larvae, this sclerite can be more prominent and more elongated as, for example, in larvae of the group Nevrorthidae (recently summarised in^41^). Even more extremely elongated sclerotised necks are known in larvae of thread-winged lacewings (Crocinae; recently summarised in^38^). Also here fossil specimens are known^16,38^, but the most extreme case of an elongated neck region has been recognised in an extant larva (Fig. 3F;^27^).

### Extreme morphologies

In lacewing larvae, many morphological structures appear to reach quite the extremes. More precisely, they are clearly more extreme in many of these structures than any other holometabolan larva.

It has been argued that Neuroptera was part of the early diversification of Holometabola^42^. This may be seen as a reason for this diversity of more extreme forms. Yet, this is not immediately an explanation for the extreme morphologies. If this would be a strong argument, we should see comparably extreme morphologies in some of the other lineages, at least among their early representatives. Also given the shear amount of species in groups such as Coleoptera, we could expect, just by the virtue of the probability, even more extreme forms. However, this seems not to be the case for most structures, with the exception of antenna length in some larvae of Scirtiidae^31^.

We can only assume that there should be a certain gene regulatory basis for the high variability of lacewing larvae. It is to be expected that holometabolan larvae strongly regulate the distal-less expression^43–45^, leading to rather short appendages in most of them.

In lacewing larvae, this is different. We can speculate which factors might have led to this difference:

1. Lacewing larvae may have acquired a de novo possibility for a stronger distal-less expression (in comparison to other holometabolan larvae).
2. Lacewing larvae may have evolved a regulation of the distal-less regulator present in other holometabolan larvae.
3. There may have been a heterochronic shift allowing larvae to “use” regulators usually only present during late metamorphosis, by this allowing the larvae to reach structure lengths otherwise only present in adults.

Unfortunately, we so far lack any type of molecular developmental data on lacewings. Even though the modern forms are less extreme than their Cretaceous counterparts in many aspects, evo-devo investigations could at least provide a basis for a better-founded discussion.

It is also worth noting that the most extreme forms are all from Cretaceous ambers, i.e., now extinct. The absence of similarly extreme forms in this lineage today, and also in all other lineages of Holometabola, could indicate that there is a strong selection against such forms. Therefore, we might be dealing with the example of the “push of the past” effect, when surviving morphologies tend to be overrepresented in the geological record^46^. Yet, the fossils clearly demonstrate that these are no principle constraints, neither functionally nor from the regulatory and developmental side. In other words, these Cretaceous larvae are strong indicators that the principle larval holometabolan body organisation has even larger potential for diversification than we see in the modern fauna.

## Conclusions and outlook

Larval forms of Holometabola represent a major share of diversity, concerning form and function. In the current time of biodiversity loss, it should be valuable to overcome the “adult paradigm”^47^ and consider the importance of immature forms also for improving conservation biology and the protection of ecosystems. It still seems that larvae are considered a scientifically not so important life phase as taxonomical approaches are more challenging, if not impossible to perform with such forms. Erecting new species based on larvae is a challenging and in some cases questionable strategy. All this leads to a strong ignorance of larval forms, especially of fossil larvae. Luckily, this seems to be changing as demonstrated by publications based on exceptional fossil larvae^11,12,15,16,18,28,36,37,39,40,48,49^. The specimens presented here likewise show that there is more to understand than just finding new species. They demonstrate, again, that considering only modern-day fauna will lead to the impression that certain morphological or developmental constraints exist, but which are in fact only artefacts due to “filtering history”. Evolutionary biology is a historical science; we therefore always should also look back in time to potentially discover spectacular finds.

## Material and methods

### Investigated specimens

Specimens for this study came from two collections. PED 0088, PED 0112, PED 0188, PED 0316, and PED 0361 came from the Palaeo-Evo-Devo Research Group Collection of Arthropods at the Ludwig-Maximilians-Universität München. They were acquired legally from various traders at ebay.com (burmitefossil, burmite-miner, macro-cretaceous). BUB 3133 and BUB 3348a came from the collection of one of the authors (PM). All described specimens are inclusions in Cretaceous Kachin amber from the Hukawng Valley, Myanmar^50^.

### Image acquisition and processing

The specimens were documented with a Keyence VHX-6000 digital microscope equipped with a 20–2000 × objective. The images were recorded under cross-polarised light to avoid reflections^51^ or under low-angle ring light^52^. They were partly recorded with HDR (high dynamic range;^53^) and with black and white background. To overcome limitations in depth of field, several images along the z-axis were recorded and fused to a consistently sharp image. To generate high-resolution images with all details visible, several image stacks along the x–y-axis were recorded and finally stitched to a panorama image^54^. Image stacking (fusion) and stitching (merging) was performed with the built-in software of the microscope. Subsequent processing of the images was performed with Adobe Photoshop CS2. The drawings of the specimens were performed with Adobe Illustrator CS2.

## Data Availability

All data is included in this paper.
